# Maternal Nutrient Restriction Alters Ca^2+^ Handling Properties and Contractile Function of Isolated Left Ventricle Bundles in Male But Not Female Juvenile Rats

**DOI:** 10.1371/journal.pone.0138388

**Published:** 2015-09-25

**Authors:** Thomas J. Harvey, Robyn M. Murphy, Janna L. Morrison, Giuseppe S. Posterino

**Affiliations:** 1 Department of Physiology, Anatomy and Microbiology, La Trobe University, Melbourne, Vic, Australia; 2 Department of Biochemistry and Genetics, La Trobe Institute for Molecular Science, La Trobe University, Melbourne, Vic, Australia; 3 School of Pharmacy and Medical Science, Sansom Institute for Health Research, University of South Australia, Adelaide, Australia; The University of Manchester, UNITED KINGDOM

## Abstract

Intrauterine growth restriction (IUGR), defined as a birth weight below the 10th centile, may be caused by maternal undernutrition, with evidence that IUGR offspring have an increased risk of cardiovascular disease (CVD) in adulthood. Calcium ions (Ca^2+^) are an integral messenger for several steps associated with excitation-contraction coupling (ECC); the cascade of events from the initiation of an action potential at the surface membrane, to contraction of the cardiomyocyte. Any changes in Ca^2+^ storage and release from the sarcoplasmic reticulum (SR), or sensitivity of the contractile apparatus to Ca^2+^ may underlie the mechanism linking IUGR to an increased risk of CVD. This study aimed to explore the effects of maternal nutrient restriction on cardiac function, including Ca^2+^ handling by the SR and force development by the contractile apparatus. Juvenile Long Evans hooded rats born to Control (C) and nutrient restricted (NR) dams were anaesthetized for collection of the heart at 10–12 weeks of age. Left ventricular bundles from male NR offspring displayed increased maximum Ca^2+^-activated force, and decreased protein content of troponin I (cTnI) compared to C males. Furthermore, male NR offspring showed a reduction in rate of rise of the caffeine-induced Ca^2+^ force response and a decrease in the protein content of ryanodine receptor (RYR2). These physiological and biochemical findings observed in males were not evident in female offspring. These findings illustrate a sex-specific effect of maternal NR on cardiac development, and also highlight a possible mechanism for the development of hypertension and hypertrophy in male NR offspring.

## Introduction

Intrauterine growth restriction (IUGR) is defined as a birth weight less than the 10^th^ centile for gestational age [1]. IUGR may be caused by a range of maternal, placental and fetal complications [2,3], making IUGR a significant health issue globally, occurring in 16.4% of births in the developing world [4] and 7–11% in developed countries [5]. Epidemiological studies by the late Sir David Barker found that small babies have an increased risk of dying from coronary heart disease [6], and this has been confirmed by a range of studies showing that small size at birth increases the risk of hypertension [7], left ventricular hypertrophy [8], and death from cardiovascular disease [9].

In an attempt to understand the basis of the increased risk of disease described above, several previous studies have examined the effects of IUGR on the contractility of the heart. These studies have predominately examined whole heart function from IUGR animals (e.g. left ventricular pressure and/or mean arterial pressure) which have typically found both increased ventricular pressure [10–14] and decreased stroke volume [15] implying changes to some step(s) within the Excitation-Contraction Coupling (ECC) cascade. ECC is a series of physiological processes that starts with the arrival of an action potential at the surface membrane of the cardiomyocyte, and ends with the generation of force at the level of the myofilaments [16]. Briefly, the action potential depolarizes the surface and t-tubular membranes leading to activation of voltage-dependent Ca^2+^ channels, which leads to an influx of Ca^2+^ from the extracellular environment. This Ca^2+^ then directly activates the cardiac isoform of the Ryanodine receptor (RyR2), which in turn results in the release of Ca^2+^ from the sarcoplasmic reticulum (SR). Consequently, the cytoplasmic [Ca^2+^] increases to the extent that Ca^2+^ is able to bind to the contractile apparatus leading to the production of force. At the end of the depolarization phase the SR rapidly re-accumulates Ca^2+^ via the SR Ca^2+^-ATPases (SERCA; of which the cardiac isoform, SERCA2a predominates) resulting in relaxation of the cardiomyocyte.

Using a global maternal nutrient restriction (NR) model [11] in Wistar Kyoto rats to induce IUGR, and using bundles of chemically skinned left ventricular myocytes, we directly examined for the first time the effects of IUGR on ECC, specifically examining both SR Ca^2+^ handling and myofilament contractility in both male and female offspring. Furthermore, we have measured relative protein abundance and phosphorylation of key proteins related to the physiological processes associated with these steps in the ECC cascade.

## Materials and Methods

### Ethical Approval

All experimental protocols within this study were approved by the Animal Ethics committee La Trobe University and comply with the National Health and Medical Research Council: Guidelines to promote the wellbeing of animals used for scientific purposes.

### Animal model

Long Evans hooded (LE) rat dams (n = 10) were housed at 22°C on a 12:12hr light/dark cycle, with *ad libitum* access to standard rat chow. Dams were mated overnight with LE males and mating confirmed by the presence of a vaginal plug on the subsequent morning. Following confirmation of mating, dams were housed individually, with food intake and body weight measured daily throughout pregnancy. At day 14 of pregnancy, 5 dams were randomly selected to undergo a 60% NR diet (based on individual dam average daily food intake during the first 14 days) for the remaining trimester of gestation. This model has previously shown to produce both morphological and physiological changes to the heart [11]. The remaining 5 dams continued with *ad libitum* access to food. At this stage, NR dams gained less weight than Control dams. Immediately following parturition, NR dams were returned to *ad libitum* food access.

Offspring were weighed on day of birth, and remained with mothers until weaning at 4 weeks. From each litter, male (n = 2) and female (n = 2) offspring were used for physiological and biochemical experiments at 10–12 weeks of age. Offspring were deeply anesthetized via isoflurane (4% v/v) inhalation and hearts rapidly excised, weighed, and placed in a Sylgard coated petri dish containing standard physiological solution containing (mM): HEPES, 7.4; Na^+^, 144, Cl^-^, 152; Mg^2+^, 1.2; Ca^2+^, 2.5; Glucose, 5.6 at pH 7.4, with constant O_2_ aeration. Under a dissecting microscope (SM71000, Nikon, Japan), the left ventricle was exposed by cutting through the aorta to the apex of the heart with dissecting scissors, and then pinned out to expose the inner ventricular wall. Bundles of left ventricular myocytes (200–300 μm in diameter) were isolated for functional analysis with the use of fine forceps and a 27G needle utilized as a micro-scalpel. Isolated bundles were tied at both ends with fine grade nylon suture thread (10/0) and mounted between a fixed pair of forceps and a force transducer (AE800, Memscap, Norway). Bundle length was set at 120% of resting length (this produced peak maximum Ca^2+^-activated force and equates to a sarcomere length of 2.2–2.3 μm [17] and the cross sectional area (CSA) was then calculated by taking the average of three diameter measurements along the length of the mounted tissue.

### SR Ca^2+^-handling experiments

SR Ca^2+^ handling broadly describes the ability of the SR to both store Ca^2+^, via the activity of the SERCA pumps and release Ca^2+^, via the activity of the RyR2. In order to obtain qualitative measurements of both SR Ca^2+^ release and SR Ca^2+^ content (which is a function of the net difference between Ca^2+^ leak through RyR2 and Ca^2+^ uptake via SERCA) we used the caffeine-induced force response as a measure of SR Ca^2+^ handling. Caffeine has been used extensively to directly stimulate SR Ca^2+^ release via activation of the RyR2 in both skinned skeletal and cardiac muscle preparations [18–24]. An estimation of the ability of the RyR2 to activate and release Ca^2+^ from the SR can be derived from measuring the rate of force development of the caffeine-induced force response (see below; [25] The area of the caffeine-induced force transient also provides a relative measure of the SR Ca^2+^ content [20].

To examine the properties of the SR of cardiomyocyte bundles, it was necessary to perforate (chemically skin) the sarcolemma of cells without damaging the SR membranes. This was achieved by using a cardiomyocyte chemical skinning procedure described previously in rat cardiac muscle [22,26]. Briefly, bundles were first exposed to a weakly buffered “skinning solution” (Table 1) containing 50 μg/ml of Saponin for 25 min. Saponin has been extensively used to preferentially permeate the surface membrane of muscle cells without affecting the integrity of the SR [22,26]. Ca^2+^ uptake and release as well as the SR Ca^2+^ content were examined before the specific contractile apparatus properties, which required more complete skinning using Triton X-100 (see later).

**Table 1:** Intracellular solutions used for skinned bundle preparations.

	A	B	C	Skinning	Load	Release
HEPES	90mM	90mM	90mM	90mM	90mM	90mM
Mg^2+^	8.6mM	10.3mM	8.12mM	8.5mM	8.6mM	8.6mM
EGTA	50μM	50mM	50mM	125μM	125μM	0.5mM
HDTA^2-^	50mM	-	-	50mM	50mM	50mM
Ca^2+^ total	-	-	48.5mM	-	100μM	-
ATP	8mM	8mM	8mM	8mM	8mM	8mM
CrP	10mM	10mM	10mM	10mM	10mM	10mM
Saponin	-	-	-	50μg/ml	-	-
Caffeine	-	-	-	-	-	30mM
pH	7.1	7.1	7.1	7.1	7.1	7.1

All skinned bundle solutions contained a free [Mg2+] of 1mM and an osmolality of 295 ± 5 mosmols kg^-1^. Creatine phosphate (CrP). Triton X-100 (2%) added to solution B when bundles were further skinned.

**Fig 1:**
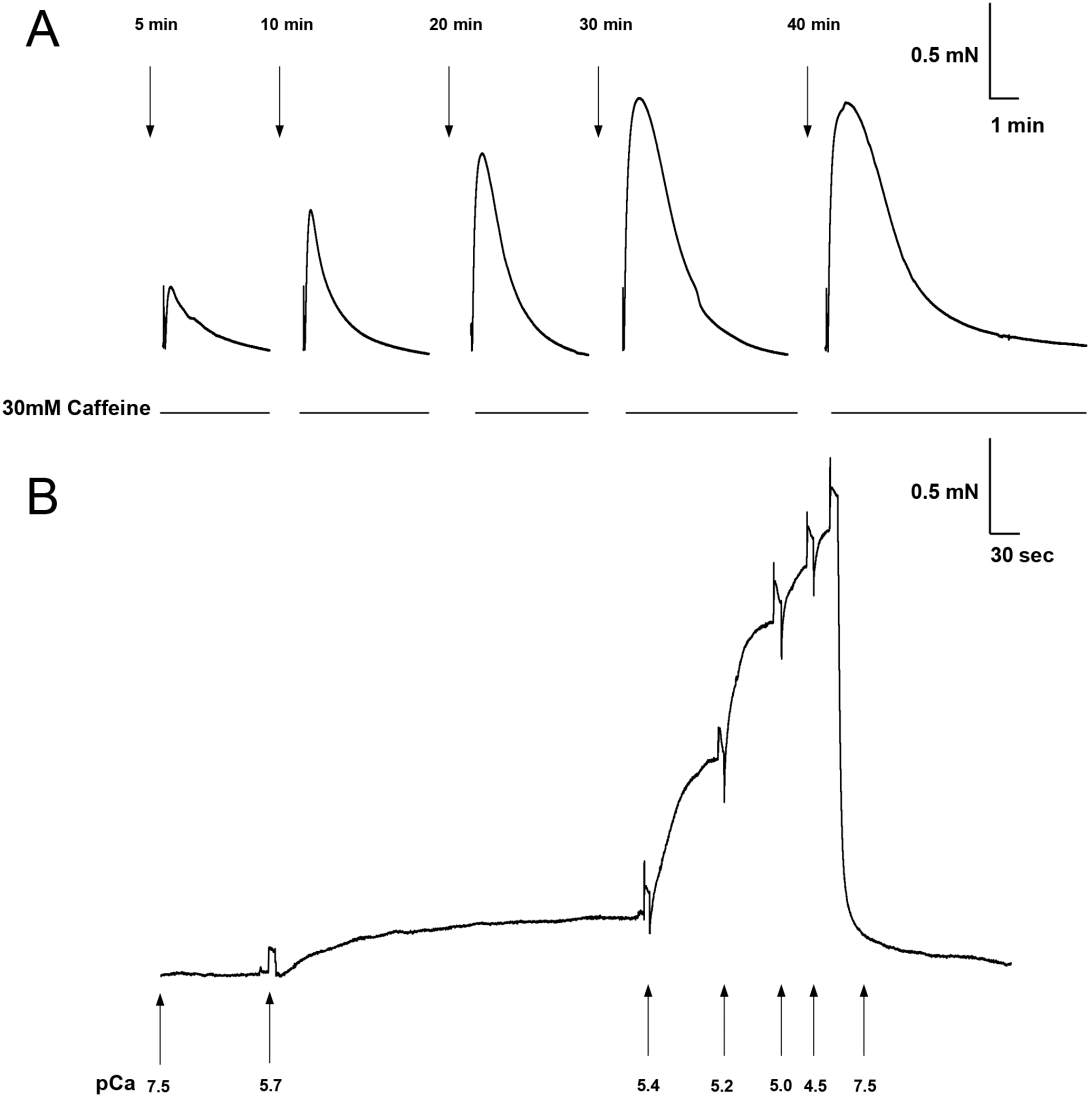
Example recordings of both caffeine-induced and Ca^2+^-activated force responses from a single cardiomyocyte bundle. (A) After skinning with 50ug.ml^-1^ saponin for 25 mins, a bundle was exposed to a load /release protocol (see methods) to ascertain the SR loading and Ca^2+^ storage properties. From left to right, the trace shows force responses to 30mM caffeine (solid line) after various load times (downward arrows). (B) The same bundle was skinned with Triton X-100 for 20 min, and the Ca^2+^ sensitivity and maximum Ca^2+^-activated force responses were then determined by exposing the bundles to heavily buffered Ca-EGTA solutions with increasing free [Ca^2+^] (pCa: 7.5, 5.7, 5.4, 5.2, 5.0, 4.5 as indicated by the upward arrows, respectively).

Skinned cardiomyocyte bundles were then washed in solution A (Table 1) to remove Saponin. The SR of bundles was then completely depleted of stored Ca^2+^ by exposing bundles to a K-HDTA solution which contained 30mM caffeine and 0.5mM EGTA (release solution) [20]. Cardiomyocyte bundles were then exposed to a Ca^2+^ load solution for time increments between 5 and 40 mins. (Fig 1A). The range of SR Ca^2+^ load times was intentionally long to examine a wide range of SR Ca^2+^-content. The slow rate of “net” SR Ca^2+^ loading observed simply reflected the difference in both Ca^2+^ leak from the SR via RYR2 (which is expected given the use of a weakly buffered Ca^2+^ solution (free [Ca^2+^] of 1μM (pCa 6.0)) confirmed with an Orion Ca^2+^-sensitive electrode (Thermo Electron) and is consistent with previous studies [22,23,26]) and Ca^2+^ uptake via SERCA. The load solution was made by mixing proportions of solution A with more heavily buffered EGTA (solution B) and Ca^2+^-EGTA (solution C) together (Table 1). Between each load period, bundles were briefly equilibrated (30s) in solution A, containing 0.5mM EGTA, to stop further SR loading before bundles were again exposed to the release solution. The area (time integral) of the caffeine-induced force response (Fig 1A) was measured in this study as it provides an accurate, qualitative estimate, of total SR Ca^2+^ content [20]. The area of each caffeine-induced force response was measured and then normalized to the maximum Ca^2+^-activated force response recorded in each bundle at the end of the experiment to allow comparison between bundles of different diameters. Because the area of a given force response is an estimate of the amount of SR Ca^2+^ loaded [20], it is also consequently an indirect estimate of the extent at which the SR accumulates Ca^2+^ via SERCA2a.

In order to estimate the activity of the RyR2, the rate of rise of the caffeine-induced force response was measured [25]. Caffeine specifically triggers Ca^2+^ release from the SR by directly activating the RyR2 [27], allowing one to obtain an estimate of the RyR2 activity by examining the rate at which the caffeine-induced force response develops using the most linear phase of the force response (10–50% of peak force). These rate values were then normalized to the maximum Ca^2+^-activated force for a given bundle to standardize for bundle diameter differences, and any changes in maximum Ca^2+^-activated force that may occur between treatments (C vs NR).

### Determination of the Ca^2+^ sensitivity and maximum Ca^2+^-activated force

In order to ascertain the properties of the contractile apparatus specifically, the same cardiomyocyte bundles used to measure the SR Ca^2+^ handling properties (described above) were subsequently further chemically skinned using Triton X-100 (2% v/v) in solution B for 20 mins, to destroy all membranous compartments. Bundles were then washed for 5 mins in solution B to remove all traces of Triton X-100.

Following Triton X-100 treatment, bundles were exposed to a pre-contraction solution (Solution A, containing 0.5 mM EGTA) for 2 mins and then exposed to a series of heavily buffered Ca^2+^-EGTA solutions containing increasing levels of free [Ca^2+^], made by mixing appropriate volumes of Solution B and solution C together, until the maximum Ca^2+^-activated force was achieved (Fig 1B). Bundles were then fully relaxed in solution B (which contains 50 mM EGTA and completely chelates all the Ca^2+^) and the procedure repeated. The average of each comparable force response was taken from the two treatment runs. Submaximal force responses were normalized to the maximum Ca^2+^-activated force and plotted against the-log free [Ca^2+^] (pCa). Non-linear regression, specifically one-phase exponential association, was used to fit the normalized data of each bundle, allowing for the Ca^2+^ sensitivity to be ascertained. The pCa generating 50% force (pCa_50_) and the Hill coefficient was measured from fitted curves in each bundle and averaged. The maximum Ca^2+^-activated force was measured by calculating the force (mN) per CSA (*Area = πr* ^*2*^) of the bundle.

### Biochemical analysis of left ventricular proteins

A further 18 Control and 18 NR offspring were utilized for biochemical analysis using a similar protocol to that described previously [28]. Briefly, samples of left ventricle (~10 mg) were weighed and homogenized in solution B (Table 1) containing 5 mM NEM, yielding a dilution of 50 μg.μl^-1^. 50 μl aliquots of homogenates were diluted with 25 μl 3x SDS solution (0.125 M Tris-Cl, 4% SDS, 10% glycerol, 4 M urea, 10% mercaptoethanol, 0.001% bromophenol blue, pH 6.8) and then further diluted to 2.5 μg wet weight.μl^-1^. Ca^2+^ handling proteins associated with both the uptake and storage of Ca^2+^ within the SR, namely calsequestrin (CSQ2) RyR2, SERCA2a, and contractile proteins responsible for force production, namely troponin I (cTnI), tropomyosin (Tm), troponin T (cTnT) and troponin C (TnC) were analyzed by Western blotting. Briefly, 25 μg wet weight of each sample was loaded onto 4–15% Criterion Stain Free™ SDS-PAGE gels (BioRad, Hercules, CA, USA), proteins were separated (200 V for 45 min), and Stain Free images captured using Stain Free™ Imaging System (Bio-Rad) for total protein quantification. Proteins were then transferred to nitrocellulose membranes (100V for 30 min). Following a series of rinses, membranes were immersed in antibody extender solution (Miser^TM^, Thermo Scientific, USA) for 10 min, and rinsed. Membranes were washed in a blocking buffer containing 5% skim milk in phosphate-buffered saline with 0.025% Tween (PBST) for 2 hr at RT, and then exposed overnight at 7°C to rabbit monoclonal anti-SERCA2a, the first of six primary antibodies to be investigated (see Table 2 for full antibody details; membranes were cut at 72 kDa and 34 kDa following SERCA2a analysis to allow for multiple antibody exposures).

**Table 2:** Antibodies used for Western blot analysis.

Antibody	Catalogue and supplier	Dilution	MW (kDa)
*Primary*			
Rabbit monoclonal anti-SERCA2a	A010-20, Badrilla	1:5000	100
Rabbit monoclonal anti-CSQ2	Ab3156, Abcam	1:1000	55
Mouse monoclonal anti-cTnI	TI-1, DSHB	1:500	29
Rabbit monoclonal anti-TnC	SC-20642, Santa Cruz	1:400	20
Mouse monocloanal anti-RYR2	Ab2827, Abcam	1:1000	565
Rabbit polyclonal anti-phospho-cTnI (Ser23/24)	4004, Cell Signalling Technology	1:1000	29
*Secondary*			
Goat anti-mouse IgG-horse radish peroxidase (HRP)	31430, Pierce	1:20000	
Goat anti-rabbit IgG-HRP	31460, Pierce	1:60000	

Five-point calibration curves were included in each gel, using a mixture of all samples (μg muscle: 3, 6, 12, 24 and 37), as previously described [29,30]. This method was used to identify the detectable signal range, and to assess the fidelity of protein transfer from gel to membrane. Linear regression of calibration curves indicated signal intensity was proportional to the amount of protein loaded. Additionally all samples loaded fell within the detectable range, allowing for direct comparison between samples of varying protein content.Following a series of washes in blocking buffer, membranes were exposed for 1 hr at RT to either goat anti-rabbit IgG-HRP, or goat anti-mouse IgG-HRP depending on primary antibody origin. Membranes went through a series of washes in 1xTBST, and chemiluminescent images were collected following exposure to SuperSignal West Femto (Pierce Thermoscientific, Rockford, IL, USA) using a charge-coupled device (CCD) camera attached to a ChemiDoc MP (Bio-Rad) and using Image Lab software (Bio-Rad). Band densities of the proteins of interest were normalized to total protein content for a given sample. cTnI content was measured as the total of two bands, a primary protein of ~29 kDa, and a secondary proteolytic fragment of ~25 kDa. Phosphorylation of cTnI at Ser 23/24 was only detected in the non-proteolyzed form, and therefore was normalized to the 29 kDa band density.

### Statistical analysis

Data were analyzed with a combination of Prism 5 (GraphPad), LabChart 7 (ADInstruments) and SPSS 19 (IBM). G*Power 3.1.2 was utilized for post hoc power analysis. Mean physiological data were analyzed with ANCOVA (offspring nested within dams, dams within treatments). Linear regression was employed to analyze SR Ca^2+^ content and rate of release data. As no grouping effects were evident for any physiological parameters investigated, biochemical data were analyzed using Student’s independent samples t-tests in samples from each sex. All findings were deemed statistically significant at *P* < 0.05.

## Results

### Nutrient restriction reduced maternal weight gain during third trimester and produced offspring below the 10^th^ percentile of controls

**Fig 2:**
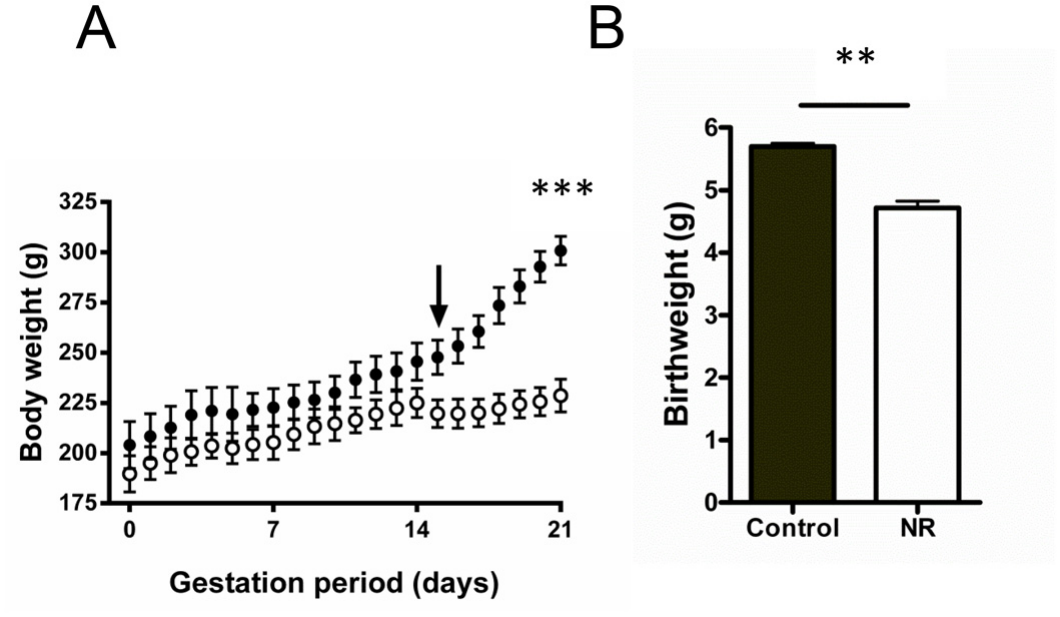
Maternal weight gain during pregnancy and subsequent birth weight of pups. (A) Maternal weight gain is shown for both Control (●, n = 5) and nutrient restricted (○, n = 5) groups over the course of gestation. Nutrient restriction was induced at day 14 (downward arrow) until parturition at day 21. All data are mean ± SEM. (Student’s t-test, ***P <0.001). (B) Mean birth weight (+SEM) for Control (n = 51) and nutrient restricted (n = 52) pups. Nutrient restricted pups were found to be significantly smaller than controls (ANCOVA, C: 5.61 g ± 0.05 Vs. NR: 4.59 g ± 0.07; *F* (1, 8) = 15.724, **P < 0.01). Nested model analysis suggested a large grouping effect associated with the intrauterine environment from which litters of pups were derived (ANCOVA, *F* (8, 93) = 17.193, **P < 0.01). Birth weight distribution of control pups indicated a lower 10^th^ percentile of 5.1 g, with > 80% of NR pups falling below this weight.

Body weight (BW) of both Control and NR dams increased by ~15% during the first 2 trimesters. Following the induction of NR protocol, subsequent weight gain in NR dams was reduced, with a 3^rd^ trimester gain of ~2.8% BW, compared with 18.5% in Controls, with NR dams significantly smaller than Controls at parturition (C n = 5, 300.8g ± 7.9 vs. NR n = 5, 228.7g ± 8.1g; Student’s t test: P = 0.0002; Fig 2A).

**Fig 3:**
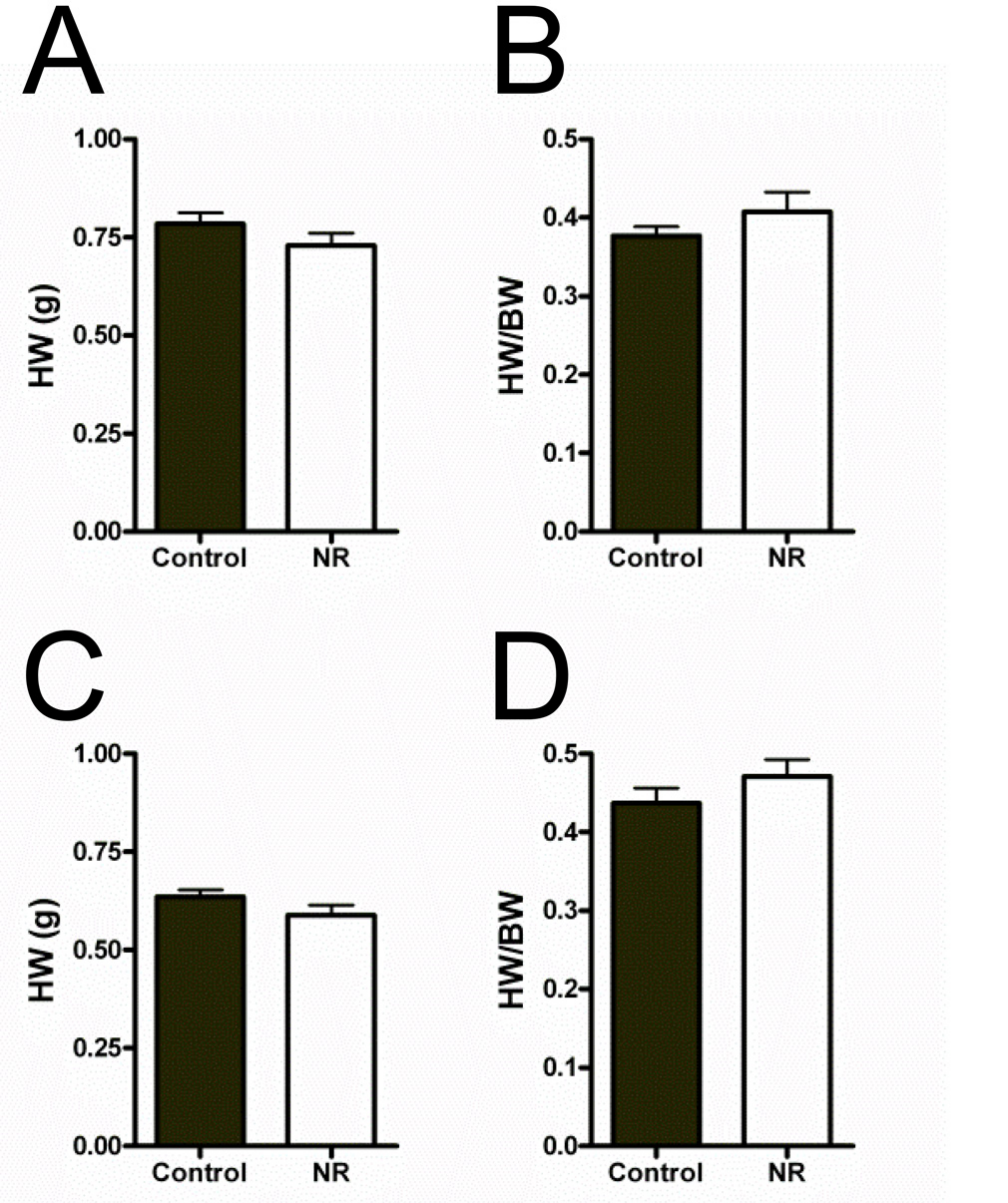
Heart weight and heart weight to bodyweight ratio in control and NR offspring used for physiological experimentation. (A-B) Data for male absolute heart weight and heart weight to bodyweight ratio. (C-D) Female absolute heart weight and heart weight to bodyweight ratio. No significant differences in heart weight, or heart weight to bodyweight ratios were observed in either sex (P>0.05).

Mean litter sizes did not differ between treatments, however, there was a significant reduction in NR offspring birth weight compared with Controls (ANCOVA, C: 5.61 g ± 0.05 Vs. NR: 4.59 g ± 0.07; *F* (1, 8) = 15.724, P = 0.004; Fig 2B), with >80% NR pups falling below the 10^th^ percentile of control pups (5.1g, as indicated by the distribution of Control offspring birth weights). At the time of physiological experimentation, 10–12 week old NR male and female offspring had no difference in heart weight (Fig 3A and 3C), or heart weight to body weight ratios (Fig 3B and 3D) compared with Controls for both sexes.

### Relative SR Ca^2+^ content was similar between treatments in both male and female offspring

**Fig 4:**
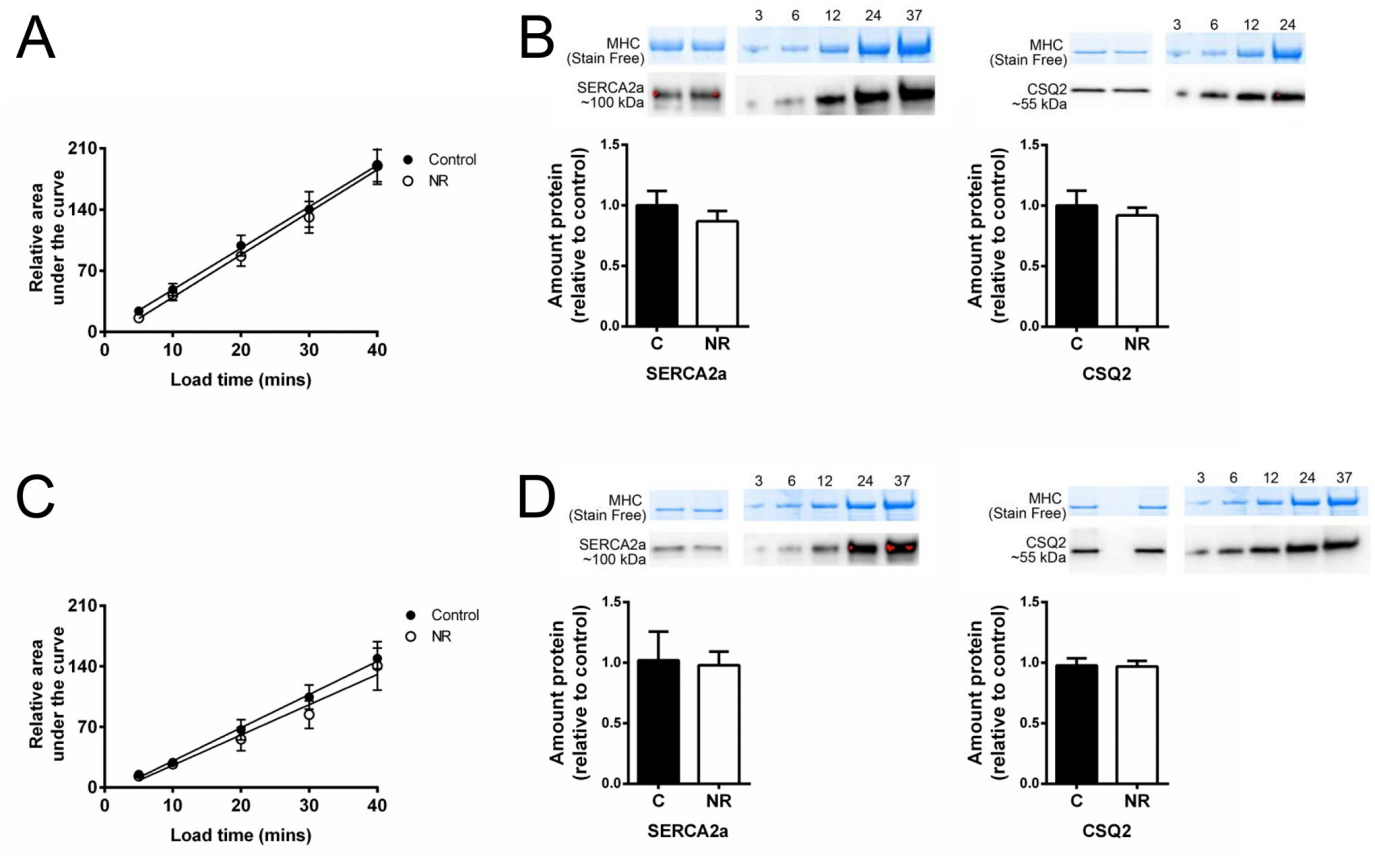
Sarcoplasmic reticulum Ca^2+^ loading properties. Sarcoplasmic reticulum Ca^2+^ loading data and relative protein content with associated standard curves for both male (A-B) and female (C-D) offspring. The relative area of individual caffeine induced force responses, indicative of the amount of Ca^**2+**^ loaded into the SR, showed no significant differences between treatments for the load times investigated (male: C, n = 10; NR, n = 10, and female: C, n = 10; NR, n = 10). Quantity of SERCA2a and CSQ2 when normalised to total protein in Stain Free gel (see methods; myosin heavy chain (MHC) indicative of amount total protein loaded) showed no significant difference between treatments for male (C n = 10, NR n = 8) or female offspring (C n = 8, NR n = 10). Values above standard curves indicate amount (μg) wet muscle loaded.

The relative SR Ca^2+^ content, ascertained by the area of the caffeine-induced force response (see Methods) showed no differences between treatments for either male or female offspring for the Ca^2+^ load times investigated (Fig 4A and 4C respectively). Relative quantification of both SERCA2a and CSQ2 was not statistically different between treatments for both male (Fig 4B) and female (Fig 4D) samples.

### Rate of Caffeine-induced Ca^2+^ release was reduced in male but not female offspring

**Fig 5:**
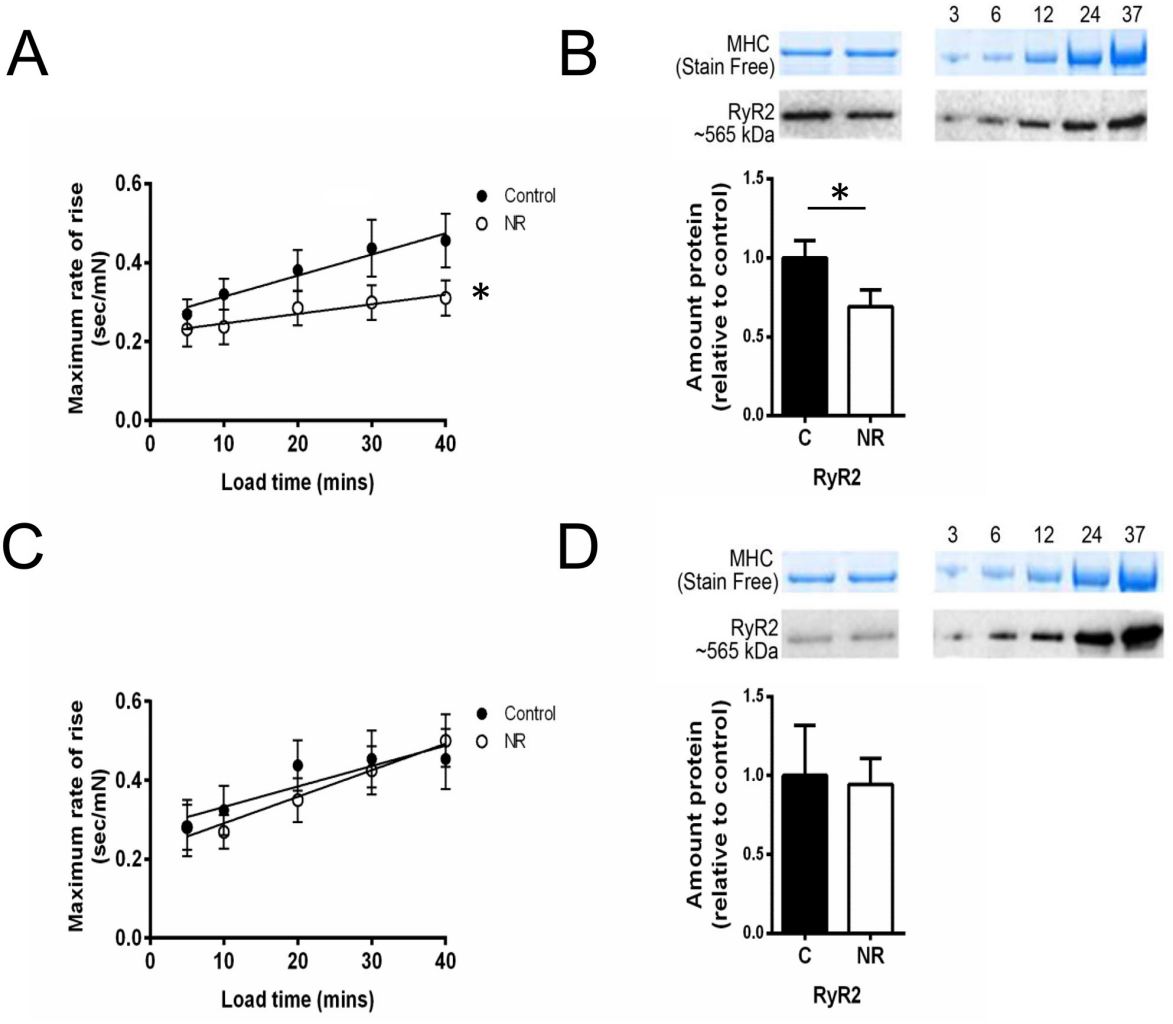
Sarcoplasmic reticulum caffeine-induced Ca^2+^ release properties. Maximum caffeine-induced Ca^2+^ release rate and relative RyR2 protein content with associated stardard curves for both male (A-B) and female (C-D) offspring. Linear regression of release rates indicated Male NR bundles had a significant decrease in maximum release rate. (*F* (1,6) = 13.74, *P<0.05). Relative amount of RyR2 protein was significantly lower in male NR samples compared with controls (C n= 10, NR n=8; Student’s t-test, *P <0.05). No differences were observed between treatments in female offspring. Values above standard curves indicate amount (μg) wet muscle loaded.

When looking at the rate of caffeine induced Ca^2+^ release specifically, by measuring the rate of rise of the caffeine induced force response (see Methods), linear regression indicated male NR offspring developed force at a significantly slower rate than Controls (*F* (1,6) = 13.74, P = 0.01; Fig 5A). The rate of caffeine-induced Ca^2+^ release was not significantly different between female Control and NR offspring (Fig 5C). Keeping in line with data obtained by physiological experiments described above concerning Ca^2+^ release, quantification of cardiac RyR2 protein revealed a 30% decrease in the relative amount from NR male samples compared to controls (C n = 10, NR n = 8. Student’s t test: P = 0.03; Fig 5B). No difference was observed between female control or NR samples (Fig 5D).

### Maximum Ca^2+^ activated force was increased in male NR offspring

**Fig 6:**
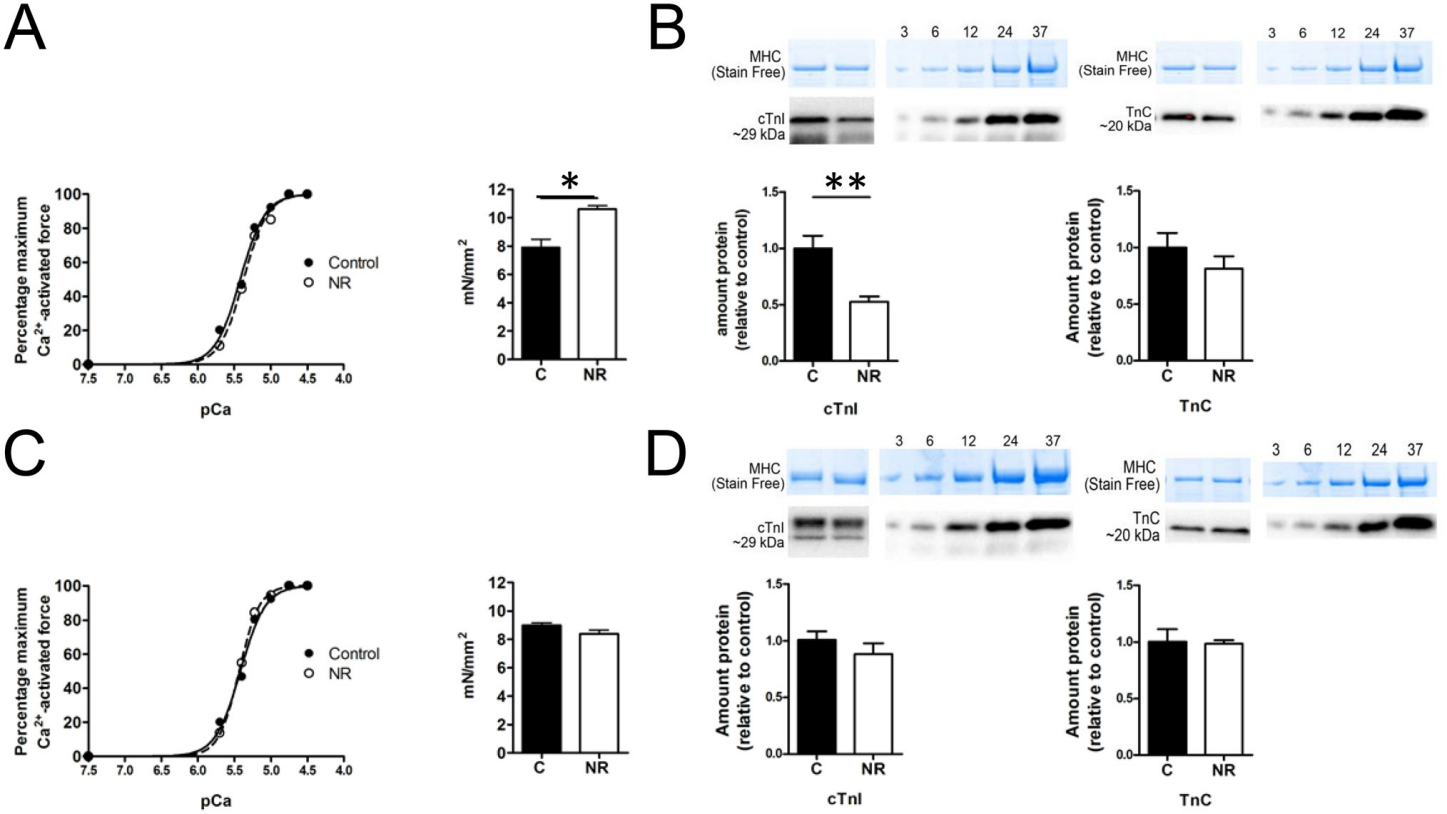
Ca^2+^ sensitivity of the contractile apparatus and maximum Ca^2+^-activated force. (A) Representative force-pCa curves from left ventricular bundles for male control and NR rats. Pooled data showed no statistical difference between control (n = 10) or NR (n = 10) in pCa_50_ or Hill coefficient. However, values of maximum Ca^2+^-activated force from the same samples showed a significant increase in maximum force produced by NR heart samples (C: 7.9 mN/mm^2^ ± 0.59 vs NR: 10.57 ± 0.25; *F* (1,8) = 8.925, *P< 0.05).(B) Quantitative analysis of integral proteins associated with the contractile apparatus in male offspring. Male C (n = 10) and NR (n = 8) showed no significant difference in the relative amount of TnC, however there was a reduction in the amount of cTnI from NR samples, showing a significant decrease compared with controls (unpaired t-test, *P<0.05). (C) Representative force-pCa curves for female control and NR rats. Pooled data showed no significant differences between control (n = 10) or NR (n = 10) for either pCa_50_ or Hill coefficient. Maximum Ca^2+^-activated force was similar between treatments. (C: 8.98 mN/mm^2^ ± 0.18 vs NR: 3.38 ± 0.27; *F* (1,8) = 2.966, P>0.05). (D) Quantitative analysis of integral proteins associated with the contractile apparatus in female offspring. No differences were observed between female C (n = 8) and NR (n = 10) samples. Proportion of cTnI proteolytic fragmentation was unchanged between treatments for either sex (see results).

Fig 6A and 6C show representative force-pCa relationships for male and female offspring, respectively. Table 3 shows the mean data of fitted pCa curves. While there was no discernable difference in Ca^2+^ sensitivity between treatments for both sexes, as indicated by pCa_50_ there was a significant increase in maximum Ca^2+^-activated force in male NR offspring, (Control, 7.9 mN/mm^2^ ± 0.59 vs. NR, 10.57 ± 0.25; *F* (1,8) = 8.925, *P* = 0.017; Fig 6A). This large increase in force production seen in NR male offspring was not observed in female NR offspring (Control, 8.98 mN/mm^2^ ± 0.18 vs. NR, 8.38 ± 0.27; *F* (1,8) = 2.966, *P*>0.05; Fig 6C).

**Table 3:** Ca^2+^ sensitivity of contractile apparatus in male and female offspring. All data expressed as mean ± SEM.

Treatment	Control	NR	ANCOVA
Male	n = 10	n = 10	
pCa_50_	5.29 ± 0.4	5.34 ± 0.05	*F* (1,8) = 0.380, P>0.05
Hill coefficient	2.08 ± 0.25	2.35 ± 0.17	*F* (1,8) = 0.092, P>0.05
Female	n = 10	n = 10	
pCa_50_	5.28 ± 0.03	5.35 ± 0.09	*F* (1,8) = 0.080, P>0.05
Hill coefficient	2.45 ± 0.25	2.46 ± 0.22	*F* (1,8) = 0.774, P>0.05

Male Control and NR samples showed no significant difference in the relative amount of the regulatory protein TnC, however the inhibitory cTnI protein showed a significant decrease compared with Controls (C n = 10, NR n = 8. Student’s t test: P = *0*.*03*; Fig 6B). There was no difference in the relative amounts of TnC or cTnI in females samples (Fig 6D). The ratio of 29 kDa to 25 kDa cTnI band densities was unaffected by treatment in either sex, suggesting no changes in the amount of cTnI proteolysis.

**Fig 7:**
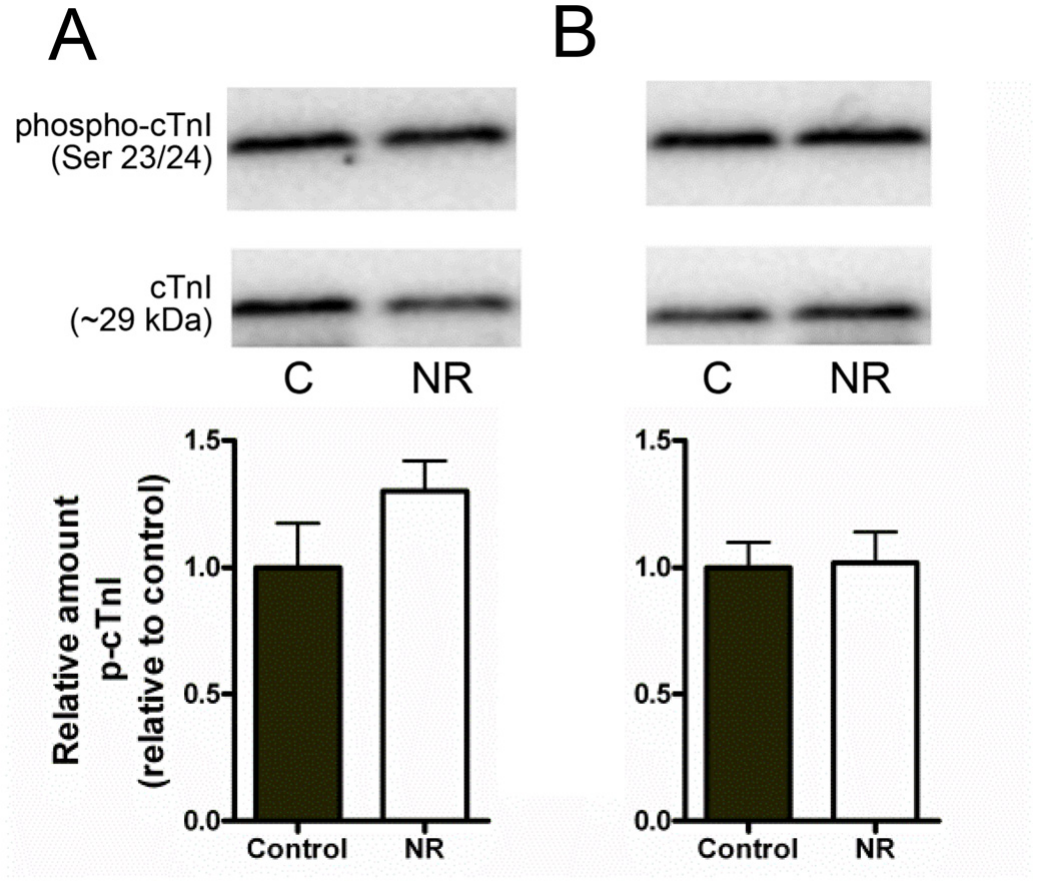
Western blot analysis of cTnI phosphorylation at Serine 23/24. Relative amount of cTnI phosphorylation at Serine 23/24 in (A) male and (B) female offspring, expressed as proportion of phosphorylation of entire (~29 kDa) cTnI protein content. No significant difference was observed between male Control (n = 10) and NR (n = 8; P = 0.09), or female Control (n = 8) and NR (n = 10; P>0.05) samples.

The relative amount of cTnI phosphorylation at Ser23/24, when normalised to the 29 kDa cTnI band for each given sample revealed no statistical difference between treatments in either male of female samples (Fig 7A and 7B).

## Discussion

Previous studies examining the effects of IUGR have alluded to changes in cardiac contractility in postnatal life without having specifically examined physiological changes to the contractile apparatus or the Ca^2+^ handling properties of the SR. In the current study, we have examined the effects of maternal NR on cardiac function in young male and female offspring. The novel findings of this study were that NR increased maximum Ca^2+^-activated force in only male offspring, and that this increase was associated with a reduction in the expression of cTnI. SR Ca^2+^ handling properties, assessed both functionally and biochemically, were also altered by NR in male offspring only, with a reduction in both the rate of rise of the caffeine-induced force response and an associated decrease in protein content of RyR2.

### Global Nutrient Restriction Model

There have been many animal models of IUGR employed in past studies (hypoxia, nutrient restriction and uterine artery ligation) and the species, including rats, sheep and guinea pigs [31–33]. Many studies examine chronic maternal hypoxia during the last trimester, which either leads to growth restriction [11] or no growth restriction [34,35]. In some cases, maternal hypoxia also results in a reduced nutrient supply typically due to a reduced food intake by the mother, making this model complicated when trying to elucidate the specific cause of growth restriction outcomes (see later). In the current study, we sought to utilize a model of IUGR that is dependent on only one factor [11]. The current study demonstrated that a 60% maternal food restriction during the third trimester of gestation produced growth restriction in offspring. Pups born to NR dams largely fell below the 10^th^ centile of normally distributed growth observed in controls. Due to natural variations in litter size, maternal body weight, and intrauterine environment, not all pups fell below the 10^th^ centile, however all pups were below the 20^th^ centile. Similarly, the reported effects of NR on heart weight have varied in the literature (see Introduction). Our results show that mean heart weight of NR animals was unchanged compared to Controls, which was consistent with the work that we model our study on [11]. Importantly, we now describe the extent of growth restriction attributable to this model, which has also previously shown to cause cardiac remodeling and increased sensitivity to ischemia/reperfusion injury [11]. Consequently, it was seen as an ideal model for investigating the effects of IUGR on ECC.

### Effects of NR on SR Ca^2+^ handling in skinned cardiomyocytes

The ability of the SR to load and store Ca^2+^, and subsequently release it, enabling relaxation and contraction respectively, is essential to maintaining contractile function in cardiomyocytes. Factors affecting the quantity of Ca^2+^ loaded and stored in the SR may include the buffering of the intracellular environment, the spatial size of the SR, and quantitative and functional properties of SERCA2a, CSQ2 and RyR2. To date no studies have investigated the properties of the SR and its associated structural and functional proteins in relation to IUGR directly. We show for the first time that the effects of global nutrient restriction leading to IUGR resulted in changes in SR properties visible only in male offspring. We show that NR did not affect the net SR Ca^2+^ content in either male or female offspring (as assessed by the area of the caffeine-induced force responses associated with different Ca^2+^ loading periods) and similarly, the expression of two key proteins that are responsible for SR Ca^2+^ uptake (SERCA2a) and SR Ca^2+^ content (CSQ2) were also unaffected. These data initially suggest that NR has little impact on both SR Ca^2+^ uptake and SR Ca^2+^ storage. However, when we examined the rate of caffeine-induced Ca^2+^ release, we note a significant decrease in the rate of rise in male offspring only. This was associated with a concomitant decrease in the protein expression of RyR2. These data suggest that NR decreases the ability of the SR to release Ca^2+^ likely through a reduction in the amount of RyR2. When we reconsider that the SR Ca^2+^ content data described above, one would expect that in NR males, the net accumulation of Ca^2+^ (which is a function of the difference between SR Ca^2+^ leak via RyR2 and SR Ca^2+^ uptake via SERCA2a) should be reduced given the reduced amount of RyR2 observed and the reduced rate of caffeine-induced Ca^2+^ release. Given that the SR Ca^2+^ contents were the same between NR and Controls it is plausible that there has been some proportional decrease in the rate of SR Ca^2+^ uptake not evident from the similar amount of SERCA2a measured between treatments. Although we did not measure the degree of phosphorylation of phospholamban (an integral regulator of the SERCA2a), it is possible that there may be differences in the amount of phosphorylation of phospholamban that would alter SERCA2a activity between treatments that could explain these data.

Although there are no prior studies that have directly examined the effects of IUGR on SR Ca^2+^ handling, the effects of maternal NR leading to IUGR in the current study bare similarities to several disease states long associated with IUGR. Previous studies in diabetic rats [36], a condition that IUGR individuals are at increased risk of [37], have shown a similar decrease in RyR2 protein content as reported in the current study. Interestingly, in human patients with end-stage ischemic cardiomyopathy, studies have shown a ~30% decrease in RyR2 mRNA expression [38,39], however when measured at the level of the protein, RyR2 abundance remained unaffected [40]. Conversely, in a study examining the effects of maternal hypoxia (which did not lead to growth restriction) on cardiac function, an increase in protein abundance of RyR2 was observed in hypoxic sheep fetuses [41].

### Effects of NR on the contractile apparatus

In our current study, we show that skinned cardiomyocyte bundles from male NR offspring produced significantly greater maximum Ca^2+^-activated force (>25%) than Controls. In skinned cardiomyocyte bundles from females, there was no difference in the maximum Ca^2+^-activated force. In addition, the Ca^2+^ sensitivity of the contractile apparatus was not different between NR and Control offspring of either sex. These data are consistent with several functional studies in whole perfused heart. When comparing both chronic hypoxia and NR during late gestation, Xu and colleagues reported an increase in LV end-diastolic pressure in 7 month old NR rats. However, in younger animals (4 months), which were around the same age as our own (10–12 weeks), NR did not produce a significant change in cardiovascular parameters measured [11]. These differences may be reconciled by a greater sensitivity of detecting changes to the functional state of the contractile apparatus in our study compared to measuring changes in ventricular pressure. Using a 70% NR model in guinea pigs, Bertram and colleagues found an increase in LV wall thickness alongside an increase in the mean arterial pressure in male offspring of dams that were NR during the last trimester [42]. Similarly, a study using male offspring from rat dams subjected to chronic hypoxia in the last trimester, showed an increased LV systolic pressure [13] however, these animals were not growth restricted at birth. More recently, a paper by Tare and colleagues showed a similar increase in LV pressure development in IUGR fetuses from male sheep [14]. Similar outcomes have also been observed previously, with increased blood pressure in male and female offspring born to placental insufficient rats [10].

There are several possible mechanisms that could explain the increased maximum Ca^2+^-activated force observed in the current study and these appear to be linked with the functional state of the troponin complex. For the first time, we show a significant reduction in cTnI levels in the NR male, but not female rats. It is becoming clearer that cTnI plays an important role in the normal functional dynamics of the heart muscle with changes in the pattern of expression during development [43,44] as well as the potential to be modulated via phosphorylation, determining the overall functional state of the contractile apparatus (for review see [45,46]). During development, there is a progressive transition from the fetal to the adult isoform of cTnI, which results in a progressive decrease in the Ca^2+^-sensitivity of the contractile apparatus [43,44]. This transition from fetal to adult troponin isoforms may also contribute to a changing pattern in the development of maximum force, particularly with the timing of the expression of TnC [44]. There is also a strong association between cTnI and maximum force development through regulation of various myofilament proteins [45,47]. Thus, a reduced cTnI observed in the current study could result in changes to maximum Ca^2+^-activated force development simply through its direct interaction with TnC—with a diminished cTnI content resulting in less inhibition of TnC leading to a more primed myofilament [48]. Alternatively, it is likely that NR animals still express some cTnI but that the adult form (measured in the current study) is replaced with the fetal slow skeletal (ss) TnI isoform (not measured), which may be characteristic of a delay in cardiomyocyte maturation that accompanies IUGR [49]. Fetal ssTnI expression would be expected to lead to an increased Ca^2+^-sensitivity [43,44], which is not evident in our current study. However, to reconcile this, we did observe a small, although not statistically significant increase in the amount of phosphorylation of Ser 23/24 on cTnI in NR males. Phosphorylation of Ser 23/24 often results in reduced Ca^2+^-sensitivity [47], and may explain the similar Ca^2+^-sensitivities in both Control and NR animals. Furthermore, previous studies in skinned rat cardiomyocytes [50] showed that PKA-induced phosphorylation of cTnI resulted in increased force production under maximal Ca^2+^ activations and this was also coupled with a reduction in Ca^2+^ sensitivity [50]. Thus, it seems possible that some physiologically relevant phosphorylation of cTnI may have occurred in our study and this has resulted in both an increased maximum force as well as a depression of the Ca^2+^ sensitivity in NR animals, which may counter an expected increase in the Ca^2+^ sensitivity if there is also a greater expression of the fetal ssTnI isoform. However, as the current study found no evidence of increased passive force (a likely scenario given the reduced inhibitory effect of cTnI), and TnC appear reduced (although again not statistically), it is possible that if one views the troponin complex as a whole, a reduction in the overall amount of the troponin sub-units may change the spatial alignment of the sarcomeres, resulting in a state of enhanced contractility.

In conclusion, we have shown sex-dependent differences in ECC parameters for the first time, with changes to both the Ca^2+^ handling properties of the SR and the functional state of the contractile apparatus in only male IUGR offspring. These data support the notion that IUGR contributes to long-term changes in the expression and function of key cardiac proteins (and the consequent physiological response of cardiomyocytes) that may contribute to an increased risk of CVD later in adult life.

## Supporting Information

S1 Dataset Excel file containing all relevant data. (XLSX)

S1 Image Lab file. Female CSQ2 Western blot.
(SCN)

S2 Image Lab file. Female cTnI Western blot.
(SCN)

S3 Image Lab file. Female p-cTnI Western blot.
(SCN)

S4 Image Lab file. Female RyR2 Western blot.
(SCN)

S5 Image Lab file. Female SERCA2a Western blot.
(SCN)

S6 Image Lab file. Female TnC Western blot.
(SCN)

S7 Image Lab file. Male CSQ2 Western Blot.
(SCN)

S8 Image Lab file. Male cTnI Western blot.
(SCN)

S9 Image Lab file. Male p-cTnI Western blot.
(SCN)

S10 Image Lab file. Male RyR2 Western blot.
(SCN)

S11 Image Lab file. Male SERCA2a Western blot.
(SCN)

S12 Image Lab file. Male TnC Western blot.
(SCN)
